# *In Vitro* Characterization of the Anti-Bacterial Activity of SQ109 against *Helicobacter pylori*

**DOI:** 10.1371/journal.pone.0068917

**Published:** 2013-07-25

**Authors:** Morris O. Makobongo, Leo Einck, Richard M. Peek, D. Scott Merrell

**Affiliations:** 1 Department of Microbiology and Immunology, F. Edward Hébert School of Medicine, Uniformed Services University of the Health Sciences, Bethesda, Maryland, United States of America; 2 Sequella, Inc., Rockville, Maryland, United States of America; 3 Division of Gastroenterology, Vanderbilt University Medical Center, Nashville, Tennessee, United States of America; Aligarh Muslim University, India

## Abstract

The most evident challenge to treatment of *Helicobacter pylori*, a bacterium responsible for gastritis, peptic ulcers and gastric cancer, is the increasing rate of resistance to all currently used therapeutic antibiotics. Thus, the development of novel therapies is urgently required. *N*-geranyl-N'-(2-adamantyl) ethane-1, 2-diamine (SQ109) is an ethylene diamine-based antitubercular drug that is currently in clinical trials for the treatment of tuberculosis (TB). Previous pharmacokinetic studies of SQ109 revealed that persistently high concentrations of SQ109 remain in the stomach 4 hours post oral administration in rats. This finding, combined with the need for new anti- *Helicobacter* therapies, prompted us to define the *in vitro* efficacy of SQ109 against *H. pylori*. Liquid broth micro-dilution was used for susceptibility studies to determine the antimicrobial activity of SQ109 against a total of 6 laboratory strains and 20 clinical isolates of *H. pylori*; the clinical isolates included a multi-drug resistant strain. All strains tested were susceptible to SQ109 with MIC and MBC ranges of 6-10 µM and 50-60 µM, respectively. SQ109 killing kinetics were concentration- and time-dependent. SQ109 killed *H. pylori* in 8-10 h at 140 µM (2MBCs) or 4-6 h at 200 µM (~3MBCs). Importantly, though the kinetics of killing were altered, SQ109 retained potent bactericidal activity against *H. pylori* at low pH. Additionally, SQ109 demonstrated robust thermal stability and was effective at killing slow growing or static bacteria. In fact, pretreatment of cultures with a bacteriostatic concentration of chloramphenicol (Cm) synergized the effects of typically bacteriostatic concentrations of SQ109 to the level of five-logs of bacterial killing. A molar-to-molar comparison of the efficacy of SQ109 as compared to metronidazole (MTZ), amoxicillin (AMX), rifampicin (RIF) and clarithromycin (CLR), revealed that SQ109 was superior to MTZ, AMX and RIF but not to CLR. Finally, the frequency of resistance to SQ109 was low and electron microscopy studies revealed that SQ109 interacted with bacterial inner membrane and cytoplasmic content(s). Collectively, our *in vitro* data demonstrate that SQ109 is an effective monotherapy against susceptible and multi-drug resistant strains of *H. pylori* and may be useful alone or in combination with other antibiotics for development as a new class of anti- *Helicobacter* drugs.

## Introduction

*H. pylori* is a spiral-shaped Gram-negative bacterium that is well adapted to infect the gastric mucosa; with approximately 50% of the world’s population colonized, *H. pylori* represents one of the most prevalent bacterial infections. Rates of colonization vary by geographic location and economic status and can be as high as 90% [1]. It is believed that *H. pylori* is typically acquired early in life by most individuals [2]. Post colonization, the bacterium can persist for months, years or decades without inducing obvious clinical symptoms [3]. However, in approximately 20% of infected individuals, *H. pylori* can induce clinical sequelae that can range from peptic ulcers to gastric cancer [4,5].

The ability to eradicate *H. pylori* infection plays a critical role in the treatment and prevention of associated gastroduodenal diseases [6,7]. Current treatment strategies typically involve 1-2 week twice-daily administration of a proton pump inhibitor (PPI) and two antibiotics, which is commonly referred to as triple therapy [8]. Though many different antibiotics have been tested for efficacy against *H. pylori*, combinations of AMX, MTZ or CLR are the antibiotics most often utilized in triple therapy. Beyond these, tetracycline (TET) and levofloxacin are gaining significant use in quadruple and “rescue” therapy regimens, respectively. Levofloxacin is especially useful following treatment failure with CLR-based treatments [9,10]. As implied by the need for quadruple and rescue therapies, *H. pylori* has developed resistance to nearly all conventional antibiotics currently used for treatment of the infection [11,12]. Indeed, the chronic nature of *H. pylori* colonization and the difficulty in eradication are responsible for the evolution of *H. pylori* treatment strategies from mono to dual to triple, and now quadruple, sequential and rescue therapies [13,14]. Adding to the complication of antibiotic resistance, prolonged periods of treatment, combined with higher doses of antibiotics and the use of multiple drugs has increased contraindications and patient non-compliance. This unfortunate cycle likely results in further selection for antibiotic resistance in *H. pylori* and allows the spread of resistant strains. Given all of these complexities, there is clearly an urgent need to develop new drugs that are effective against resistant strains and that have the ability to traverse into the gastric epithelial cells to eradicate any intracellular *H. pylori* cells that cannot be reached by other antibiotics.

Despite significant increases in emergence and spread of multi-drug resistant strains of *H. pylori* over the past 20-30 years, the development of new antibiotics has decreased alarmingly in the same period of time [15–17]. Furthermore, many researchers have switched from traditional drug search to genomic applications, which have unforeseeably taken longer to produce candidate antibiotics than originally expected [18,19]. Indeed, in the past half century only three new classes of antibiotics have entered the clinics: lipopeptides [20], oxazolidinones [21] and streptogramins [22,23]. All three of these antibiotics specifically target Gram-positive bacterial infections, which further limits the availability of effective antibiotics against Gram-negative bacteria such as *H. pylori*.

A potential antibiotic in clinical development is *N*-geranyl-N'-(2-adamantyl) ethane-1, 2-diamine (SQ109), which represents a member of a new class of small molecule ethylenediamine compounds that has anti-tubercular activity [24,25]. SQ109 has strong *in vitro* bactericidal activity against *Mycobacterium tuberculosis* and traverses host cell membranes to effectively kill *M. tuberculosis* in macrophage phagolysosomes [24]. Moreover, SQ109 has synergy with two front-line TB drugs (isoniazid and RIF) in both *in vitro* studies and in pre-clinical animal trials [26,27] and is safe and well-tolerated in humans [28,29]. SQ109 is currently in Phase 2 clinical trials for treatment of adult pulmonary TB.

Pharmacokinetic studies of SQ109 showed promising bioavailability with accumulation of high concentrations of the drug in the stomach [25,30], the natural niche of *H. pylori*. This finding, along with the fact that SQ109 has proven safe in humans led us to ask whether SQ109 showed antibacterial activity against *H. pylori*. Herein, we describe the *in vitro* characterization of SQ109 anti-*H. pylori* activity. Our data suggest that SQ109 has an interesting potential as a new therapeutic and may be suitable for development as a new antibiotic for the treatment of *H. pylori* infections.

## Materials and Methods

### SQ109, reagents and bacterial Strains

SQ109, which was originally developed and identified by combinatorial chemistry and high-throughput screening of over 63,000 library analogs, was synthesized on solid support using a novel acylation-reduction sequence as previously described [24,25], and was provided as a dry powder by Sequella, Inc. (Rockville, MD). SQ109 was dissolved in water to form a 10mg/ml stock solution, which was then stored as 250µl single use aliquots at -20^o^C. AMX, CLR and MTZ were purchased from Sigma (St. Louis, MO), and vancomycin was obtained from USB Corporation (Cleveland, OH). Each drug was reconstituted according to the manufacturer’s instructions and used at the indicated concentrations.

A total of 26 *H. pylori* strains and isolates (Table 1 and Table 2) used in the study were obtained as follows: 6 strains (G27, 7.13, HPAG, SS1, J99 and 26695) are common laboratory strains contained in our strain collection, while 20 isolates were low passage clinical isolates collected from patients at several locations. Of these 20 clinical isolates, 6 strains (K3, K93, K154, K260, K264, K266) were obtained from patients presenting for treatment at the Department of Internal Medicine at the College of Medicine of The Catholic University of Korea in Seoul, South Korea, 12 strains (B99, B105, B107, B108, B128, B129, B138, B148, B235, B240, B289, J104) were obtained from patients at Vanderbilt University Medical Center, Nashville TN, and 2 strains (USU102, USU103) were obtained from patients at the Veterans’ Bureau, VA Medical Center, Bethesda, MD. Of these strains, USU103 was obtained from a patient that failed four rounds of antibiotic treatment, including one round of quadruple therapy; antibiotic sensitivity testing showed the strain to be highly resistant to AMX and CLR (Figure S1). Thus, the strain represents a multi-drug resistant isolate.

**Table 1:** Antimicrobial Activity of SQ109 against *H. pylori* Laboratory Strains.

**Laboratory Strains**	**Reference**	**SQ109 Concentration (µM)**	
		**MIC^a^**	**MBC^b^**
G27	[69]	8-10	65-70
7.13	[70]	8-10	75-80
HPAG	[71]	8-10	70-75
SS1	[72]	10-15	65-70
J99	[73]	10-15	65-70
26695	[74]	15-20	80-100

^a^ Minimum inhibitory concentration (100.1% survival); **^b^**Minimum bactericidal concentration (99.9% killing). The data represent results from at least three independent experiments.

**Table 2:** SQ109 efficacy against *H. pylori* clinical isolates.

***H. pylori* Clinical History**		**Clinical Isolates Identification**	**SQ109 Concentration (µM)**	
**Endoscopic and Histologic Diagnosis**	**Histologic Features**		**MIC^a^**	**MBC^b^**
Gastritis	Glandular atrophy score 1 or 2	K154	6-8	50-60
		K266	8-10	50-60
		B99	5-10	50-60
		B108	8-10	60-65
		B235	8-10	60-70
		B289	15-20	50-60
Duodenitis	Glandular atrophy score 1 or 3	B107	15-20	60-65
		B240	8-10	60-65
		B148	6-8	60-65
Gastric Ulcers	Glandular atrophy score 1 or 3 Intestinal metaplasia	B105	25-30	70-80
		B128	6-8	70-80
	Multi-drug resistant strain, MDRS**^c^**	USU103	10-15	50-60
Duodenal Ulcers	Glandular atrophy score 1 or 3 Intestinal metaplasia	K93	6-8	50-60
		K264	5-10	50-60
		B129	6-8	65-70
		J104	10-15	60-70
Gastric Cancer	Adenocarcinoma	K3	5-10	50-60
		K260	5-10	65-70
		B138	6-10	70-75
		USU102	20-25	50-60

^a^ Minimum inhibitory concentration (100.1% survival); **^b^**Minimum bactericidal concentration (99.9% killing); **^c^**MDRS = multi-drug resistant strain (quadruple and sequential therapies administered and see Figure S1). The data represent results from at least three independent experiments.

### Determination of SQ109 MIC and MBC against *H. pylori*

All strains were grown at 37^o^C under micro-aerobic conditions of 5% O_2_, 10% CO_2_, and 85% N_2_ gas mixture, which was achieved using an Anoxomat instrument (MART 1598, Spiral Biotech, Norwood, MA). The 26 clinical and laboratory strains were used to determine the MIC and MBC of SQ109 by liquid broth micro-dilution as previously described [31]. Briefly, stock cultures stored at -80^°^C, were used to inoculate horse blood agar (HBA) plates. The strains were cultured for 24 h on HBA plates and then expanded and recultured for an additional 24 h before being used to inoculate Brucella broth (Neogen-Acumedia) liquid starter cultures supplemented with 10 µg/mL vancomycin (Sigma) and 10% FBS (Invitrogen Gibco-BRL, Carlsbad, CA) (complete Brucella broth medium, CBBM). The starter cultures were incubated for 16-18 h and then used to inoculate experimental cultures at an optical density at 600nm wavelength (OD_600_) of 0.05. These samples were used to perform the MIC and MBC experiments in 1 ml culture aliquots in sterile 15 mm diameter glass tubes. To identify the initial concentration ranges for MIC and MBC determination, we incubated the bacterial cells with two-fold dilutions of SQ109 that ranged from 10–200 µM. The samples were incubated with shaking at 110 rpm for 24 h. Bacterial cells incubated with PBS were included as a control to SQ109-treatment. The MIC and MBC were determined by enumeration of surviving colony forming units (CFU) on HBA plates and were expressed as percent CFU survival using the formula: % CFU Survival= (CFU_t24_/CFU_t0_)100; where CFU_t24_ is the number of CFU at the end of 24 h of culture and /CFU_t0_ is the starting CFU at the beginning of the assay. After the initial assays, susceptibility assays were next repeated 3 times with smaller concentration intervals; steps of 2-5 µM and 5-10 µM were used for the determination of specific SQ109 MIC and MBC, respectively. The lowest SQ109 concentration at which there was no evidence of growth (100.1% CFU survival) was recorded as the MIC; MBC was defined as the lowest concentration of the drug that killed 99.9% of the starting CFU (0.1% CFU survival).

Since USU103 was reportedly a multi-drug resistant isolate (A. Dubois personal communication), we sought to determine its resistance to selected conventional antibiotics often used against *H. pylori* (AMX, MTZ, CLR, and TET). Bactericidal assays were conducted in 1 ml liquid cultures as described above and strain 26695 was included for comparison. The percent survival of *H. pylori* clinical isolate, USU103 was computed against a range of concentrations of AMX (0.5-1000 µg/ml), MTZ (0.5-1000 µg/ml), CLR (0.5-100 µg/ml), and TET (0.5-100 µg/ml) by plating and CFU enumeration following culture in the presence of the indicated drug for 24 h (Figure S1).

### Determination of killing kinetics of SQ109

The time required for SQ109 to kill *H. pylori* cells was determined using time-kill curves by exposing the various strains to a range of SQ109 concentrations (10-200 µM) for up to 24 h, followed by monitoring of CFUs. Briefly, 1 ml liquid bacterial cultures were diluted to an OD_600_ of 0.05 in CBBM and exposed to SQ109. After 0, 1, 2, 4, 6, 8, 10, 20, 22 and 24 h of culture in the presence of SQ109, 10-fold serial dilutions of the samples were plated on HBA plates. The plates were incubated at 37°C for 5-6 days at which point CFU were enumerated and expressed as CFU/ml. *H. pylori* cells exposed to similar volumes of PBS (0 µM SQ109) were included as a control.

### Determination of the effects of SQ109 on slow-growing *H. pylori*

A static culture of *H. pylori* was obtained by using a pre-determined bacteriostatic concentration of Cm; treatment with 30 µM Cm inhibits *H. pylori* growth but does not kill the bacterium. In order to determine if SQ109 had the ability to kill slowly growing *H. pylori*, time kill curves were evaluated over a 24-hour period in CBBM liquid cultures as described above but with the following modifications. Briefly, a 5 ml *H. pylori* starter liquid culture was incubated with 30 µM Cm for 16 h at which point, the OD_600_ was adjusted to 0.05-0.07 and the sample was divided into four 1 ml samples. The bacterial samples were incubated with shaking with 0 µM, 10 µM, 70 µM or 140 µM SQ109 for 24 h. At 0, 2, 4, 6, 8, 10, 14, 20, 22, and 24 h, an aliquot of each sample was plated and CFU were enumerated. A sample of *H. pylori* incubated with PBS or Cm-free ethanol for 16 h and then adjusted to an OD_600_ that matched that of the bacteria cultured in the presence of Cm was included as a control. The time-kill curves of bacterial samples cultured in the presence of combinations of 30 µM Cm with or without SQ109 were compared to those of bacterial samples exposed to PBS or SQ109 alone.

### Determination of SQ109 stability under different thermal and pH conditions

SQ109 stability at low pH and following incubation at different temperatures was investigated using time-kill assays as outlined above. To characterize the impact of temperature on stability, 140 µM of SQ109 was incubated at 22^o^C, 37^o^C, 60^o^C or 95^o^C for 1 h prior to performing the time-kill assay. The samples were then cooled on ice and the killing time was determined as described above.

To examine the stability of SQ109 at low pH, the pH of CBBM was adjusted to pH 4.5 using HCl. The media was subsequently filter-sterilized to remove precipitates and was used for time-kill curve experiments as described above, with the following exceptions: *H. pylori* cells were suspended in 25 ml of pH 4.5 or pH 6.8 CBBM medium at an OD_600_ 0.05 and subsequently exposed to a final concentration of 140 µM SQ109 or PBS (0 µM). The liquid bacterial cultures were incubated with shaking for up to 24 h. The killing time for both experiments (pH and temperature), were evaluated by enumeration of CFU on HBA plates at 0, 2, 4, 6, 8, 10, 14, 20, 22, and 24 h. Additionally, for the pH stability assays, the pH was monitored across the 24 h culture period to ensure stability. Of note, to control for any effects of nutrients lost by precipitation during the adjustment of pH of the media, the experiment was repeated using pH 6.8 CBBM media whose pH was initially HCl-adjusted to pH 4.5, filtered and then readjusted back to pH 6.8 using NaOH solution. Similar results were obtained.

### Comparative efficacy of SQ109, AMX, MTZ and CLR against *H. pylori*

The antibacterial activity of SQ109 against *H. pylori* was compared to that of AMX, MTZ and CLR using time-kill assays as described above. For these assays, the *H. pylori* G27 strain was exposed to 70 µM or 140 µM SQ109, AMX, MTZ and CLR in 1 ml CBBM. Samples were withdrawn at 0, 0.5, 2, 4, 6, 8, 10, 14, 20, 22, and 24 h and the number of viable CFU determined as described above.

### Determination of frequency of resistance to SQ109

Frozen *H. pylori* strains G27, 26695 and B105 were revived on HBA plates as described above (MIC and MBC experiment). The cells were inoculated into 25 ml CBBM liquid cultures, which were then grown for 16-18 h; at this point the cultures showed an OD_600_ of 0.5-1.0. One milliliter aliquots of each culture were concentrated by centrifugation, resuspended in 200 µl of CBBM and then plated on selective HBA plates that contained 100 µM, 200 µM, or 400 µM SQ109. The total number of bacteria present in the cultures was obtained by serially diluting the starting culture and plating on SQ109-free HBA plates. CFUs appearing on the plates following incubation for 5-6 days at 37^o^C were enumerated. This entire process was repeated a total of 4 times and the frequency of SQ109-resistant mutants was determined by dividing the total number of CFU obtained on SQ109-selective medium by the total number of CFU obtained on the SQ109-free medium (wild-type) across all of the plating experiments.

To monitor the stability of resistance, and to determine whether resistant colonies might in fact represent persistent strains and not resistant strains, 3-5 individual SQ109-resistant mutant colonies were randomly picked, streaked and expanded on SQ109-free HBA plates to create stock cultures. These variants were then expanded from the freezer and serially passaged as lawns on SQ109-free plates a total of 5 times; each plate was incubated for 24 hours, the resulting lawn was swabbed and the cells used to lawn a new plate. The MIC and MBC of these isolates to SQ109 was then determined and compared to the parental/wild-type strain.

### SQ109 membrane permeation assay using 1-N-phenylnaphthylamine (NPN)-uptake

SQ109 was shown to exert its effect on *M. tuberculosis* by inhibiting the assembly of mycolic acids into the cell wall core [32]. Therefore, to begin to understand mechanism(s) of action of SQ109 against *H. pylori*, we performed membrane permeation assays as previously described [33] using NPN-uptake as a means to evaluate whether the drug targets and disrupts the bacterial membrane.

### Transmission electron microscopy (TEM) studies

TEM analysis was performed as previously described [33]. Briefly, approximately 1.5 x 10^7^ G27 bacterial cells were treated with 140 µM SQ109. Samples were taken at 2 or 8 h, washed twice in PBS and then used for TEM. Bacterial cells treated with 100 µM AMX, 20 µM C_12_K-2β_12_ (a peptide previously shown to form pores in *H. pylori* membranes [33]) or PBS were included as controls. Treated cells were resuspended and fixed in PBS containing 2% formaldehyde/2% glutaraldehyde/0.5% tannic acid (v/v/w) for 1 h at room temperature. Following fixation, the bacterial cells were thoroughly rinsed, enrobed in agarose, and trimmed into 1 mm^3^ blocks. The enrobed blocks were post-fixed with 1% osmium tetroxide in phosphate buffer, dehydrated by passage through a graduated ethanol series and embedded in Spurr’s resin (Electron Microscopy Sciences, PA) following the manufacturer’s recommendation. The blocks were trimmed and sectioned using a Reichert Jung ultra-microtome UC6. Sections measuring 70-80 nm were collected onto copper grids. Finally, the grids were successively stained with 1% uranyl acetate and Sato’s triple lead stains and subsequently examined in a FEI Tecnai T12 transmission electron microscope at an accelerating voltage of 80 kV. TEM images were examined at low (6,500x) and high (42,000x) magnifications for bacterial morphology and ultra-structure analysis, respectively. The images were acquired with an AMT XR60B digital camera (Advanced Microscopy Techniques) using AMTV600 software.

## Results

### SQ109 efficacy: Determination of MICs, MBCs and killing kinetics

SQ109 bactericidal activity against *M. tuberculosis* and the accumulation of the drug in high concentrations in the stomach after oral administration in mice and rats [25] prompted us to determine the activity of this anti-tubercular drug candidate against *H. pylori*. To determine SQ109 efficacy against the bacterium, we first evaluated the MIC and MBC against a panel of six commonly used laboratory strains. All of the laboratory strains tested were susceptible to SQ109, with MIC ranges of 8-20 µM and MBC ranges of 65-100 µM (Table 1). Among the laboratory strains, *H. pylori* strain G27 was one of the most susceptible to SQ109 antibacterial activity (MIC=8-10 µM; MBC=65-70 µM), thus G27 was used in subsequent experiments to elucidate SQ109 antimicrobial activity. In contrast, strain 26695 was the least sensitive (MIC=15-20 µM; MBC=80-100 µM). Given that these laboratory strains have been cultured for long periods of time and that *H. pylori* displays a high rate of genetic variability in culture [34,35], we next analyzed a panel of 20 low passage clinical isolates for susceptibility to SQ109. These included isolates obtained from a diverse number of possible *H. pylori*-associated diseases (Table 2). Once again, all of the strains were susceptible to SQ109, with MIC ranges of 5-30 µM and MBC ranges of 50-80 µM (Table 2). Of note, USU103, which was obtained from a gastric ulcer patient who had failed rounds of triple and quadruple therapies, was predicted to be a multi-drug resistant strain. Indeed, MIC and MBC testing of AMX, MTZ, CLR and TET against the isolate demonstrated that USU103 was resistant to AMX, CLR and MTZ (Figure S1), whereas 26695 was sensitive to all of the tested antibiotics. *En masse*, 22 of the 26 examined strains (84.6%) had MICs from 5–15 µM while 4 of 26 (15.4%) had slightly higher MIC ranges of 15-30 µM. Similarly, 20 of the 26 examined strains (76.9%) had MBC ranges of 50-70 µM and 6 of 26 (23.1%) had slightly higher MBCs of 70-100 µM. Taken together, our data suggest that SQ109 has potent antibacterial activity against all tested strains of *H. pylori* and is effective against at least one multi-drug resistant strain.

### Time kill-curve analysis demonstrates SQ109 time- and dose-dependent killing kinetics

**Figure 1:**
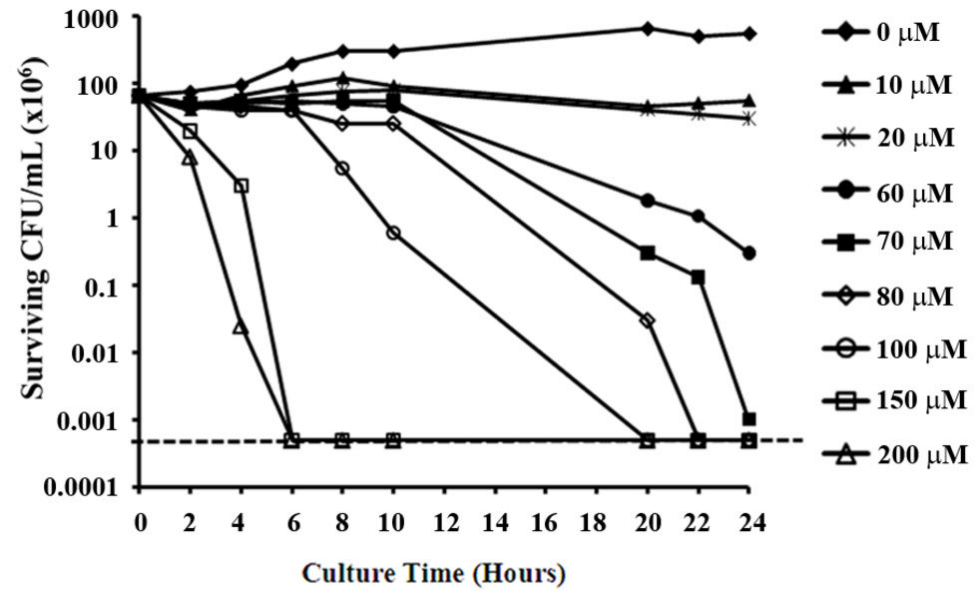
Dose-dependent killing of *H. pylori* strain G27 by SQ109. Approximately 8 x 10^7^ cells were incubated with increasing concentrations of SQ109. The cultures were monitored for 24 h and sampled at the indicated times to determine surviving CFU by plating. The horizontal dashed line indicates the limit of detection (500 bacteria). The data are representative results from five independent experiments.

The rate at which an antibiotic exerts its antibacterial activity is particularly important when considering possible treatments for *H. pylori* infection. This is due to the relatively rapid transit of gastric contents, which has the potential to reduce the exposure time of the bacteria to a particular concentration of an orally-ingested antibiotic. Therefore, to determine the rate of SQ109-dependent killing, we performed time-kill assays against strain G27. As shown in Figure 1, no colonies were recovered after 6 h of treatment with 200 µM SQ109 (approximately three-fold the MBC). Analysis of the slopes of the kill curves revealed that the time required to kill 90% of the bacterial culture could be shortened by increasing the concentration of SQ109; 90% of the G27 strain was killed in approximately 7 h after incubation with 100 µM SQ109 while the same amount of killing could be achieved in 3-4 h upon incubation with 200 µM SQ109. Similar killing kinetics were observed against strain 7.13 (data not shown). Furthermore, similar killing kinetics were also obtained when the SQ109 experiments were conducted in vancomycin-free media; thus, vancomycin in the culture media had no effect on the potency of SQ109 (data not shown). These data indicate dose-dependent and time-dependent killing of *H. pylori* by SQ109.

**Figure 2:**
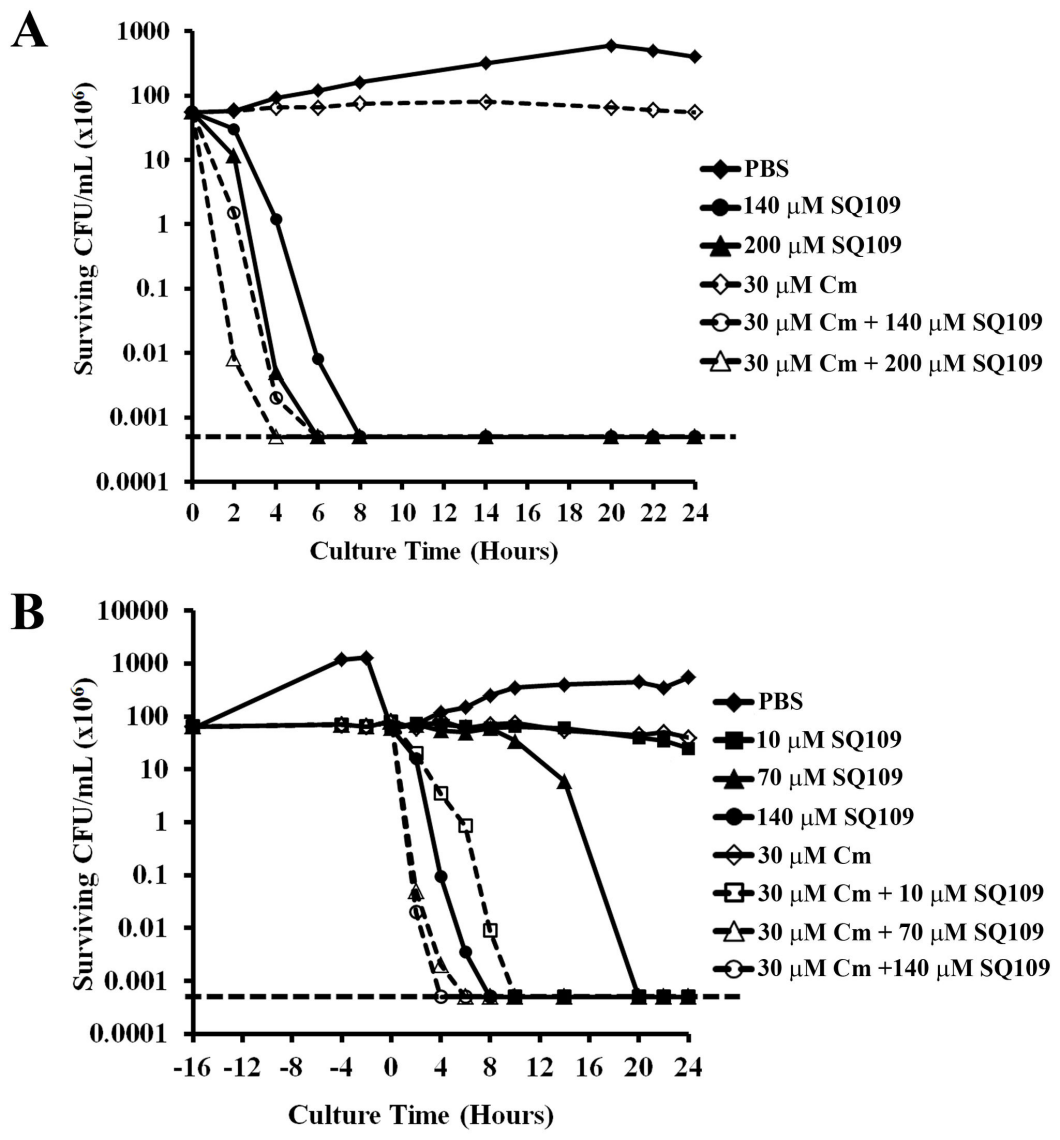
Antibacterial activity of SQ109 against slow-growing *H. pylori* and synergy with chloramphenicol, Cm. A predetermined bacteriostatic concentration of Cm (30 µM), was used to induce slow growth. Synergistic effects of SQ109 and Cm was then examined using two approaches: **A**) *H. pylori* strain G27 liquid cell cultures were treated simultaneously with 30 µM Cm and SQ109 (0 µM, 140 µM, and 200 µM) (open symbols), and the cells were monitored for 24 h, and **B**) *H. pylori* strain G27 cells were initially incubated with 30 µM Cm for 16 h in liquid culture medium before being split into multiple samples for further incubation with varying concentrations of SQ109 (0 µM, 10 µM, 70 µM or 140 µM) (open symbols) plus Cm. In both approaches, bacterial cells incubated with only PBS, SQ109 (0 µM, 10 µM, 70 µM, 140 µM or 200 µM) or Cm alone, were used as controls (closed symbols). The data are representative results from three independent experiments. The horizontal dashed line on each graph indicates the limit of detection (500 bacteria).

### SQ109 efficacy against slow-growing bacteria

*In vitro*, antimicrobial agents are often tested against exponentially growing bacteria in order to achieve maximum bactericidal effects; however, in *in vivo* environments, pathogens often experience stress and nutrient limitations that slow or prevent bacterial growth [36,37]. Thus, we sought to determine the efficacy of SQ109 against a static or slow-growing culture of *H. pylori* strain G27. Growth arrest of the culture was achieved by exposure of the bacteria to a pre-determined concentration of Cm (30 µM). As shown in Figure 2A, the addition of 30 µM Cm inhibited *H. pylori* growth, but had no effect on survival. Conversely, addition of 140 µM or 200 µM SQ109 resulted in complete killing of the culture within 8 h or 6 h, respectively. Simultaneous addition of Cm and SQ109 to the culture similarly resulted in complete killing of *H. pylori*, though the kinetics of killing were slightly faster; 6 and 4 h were required to achieve complete clearance of bacteria with 140 µM or 200 µM SQ109, respectively (Figure 2A). These results suggest that SQ109 is active against *H. pylori* regardless of the growth state of the cells. Despite these results, we next considered the fact that the simultaneous growth arrest, through the addition of Cm, might not adequately recapitulate *in vivo* growth arrest. Colonizing *H. pylori* within the stomach would likely be in a state of continuous slow or no growth well before the addition of any therapeutic agent. Thus, we next asked whether SQ109 would be effective against bacterial cells that had been in a static state for an extended period of time. To achieve this state, we treated cultures initially with Cm for 16 h before washing and exposing to SQ109, Cm or SQ109 + Cm. Moreover, in order to demonstrate possible synergy in antibacterial activity of SQ109 in combination with Cm, pre-determined bacteriostatic concentrations of the two drugs were used (10 µM SQ109 [Figure 1] and 30 µM Cm [Figure 2A]). As expected, treatment with Cm for 16 h did not significantly damage the *H. pylori* cells since removal of the drug and exposure to PBS resulted in growth (Figure 2B). Similarly, incubation of cells with similar volumes of ethanol to control for ethanol used in solubilization of Cm, resulted in a growth profile similar to cells incubated with PBS (data not shown), indicating that the small volume of solvent did not impact *H. pylori* growth or survival. Exposure of the cells to increasing concentrations of SQ109 resulted in dose-dependent killing of *H. pylori* with killing kinetics similar to those previously observed in Figure 1 (Figure 2A). Continued exposure of the cells to Cm alone resulted in bacteriostasis and no dramatic changes in number of bacterial cells. However, the antibacterial activity of SQ109 was remarkably enhanced against *H. pylori* when the cells were pretreated with Cm and Cm remained in the culture (30 µM Cm + 10 µM SQ109; [Figure 2B]). While 10 µM SQ109 alone was bacteriostatic, the bacterial samples treated with 10 µM SQ109 + 30 µM Cm resulted in complete killing within 10 h (Figure 2B), indicative of drug synergy. A similar synergy was seen with 70 µM SQ109 + 30 µM Cm, however, the synergy was not as evident at 140 µM SQ109 + 30 µM Cm. This is likely due to rapid killing already observed at the higher concentration of SQ109. Taken together, our results indicate that SQ109 is active against slow-growing bacteria, which further suggest relevance of the antibacterial activity *in vivo* where the bacteria often show slow growth. These results further suggest that synergy may be achieved in combination therapy by initial pretreatment of the bacterial infection with a bacteriostatic agent, such as Cm.

### Effect of temperature and pH on antibacterial activity of SQ109

**Figure 3:**
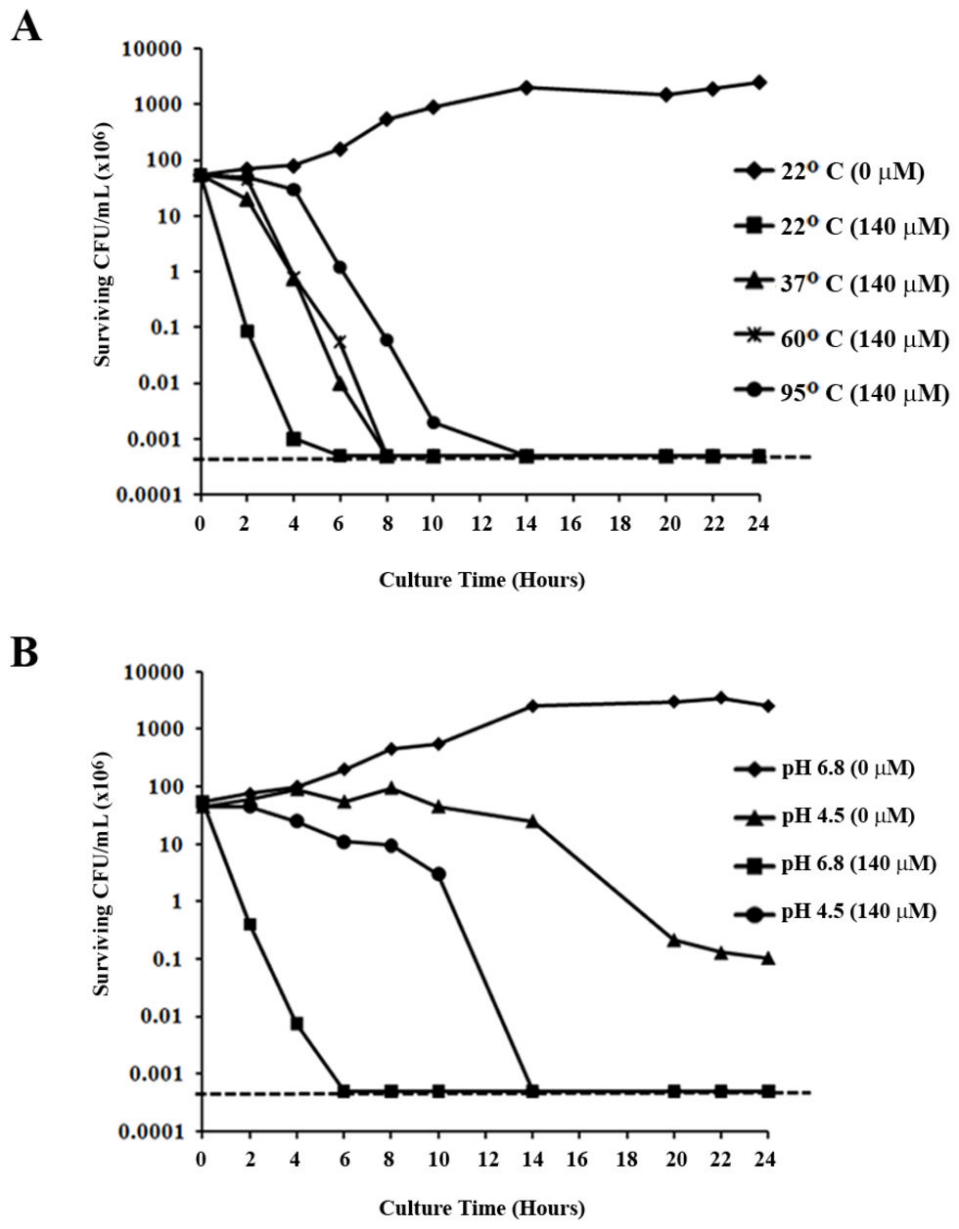
Effect of temperature and low pH on the stability and bactericidal activity of SQ109. **A**) The effect of temperature on bactericidal activity of SQ109 was examined by pre-incubation of the drug at various temperatures (22^o^C, 37^o^C, 60^o^C and 95^o^C) for 1 h prior to use in the time-kill assay. **B**) In order to determine the effect of pH on SQ109, the time-kill assays were performed in pH-adjusted culture medium. The antibacterial activity of 140µM SQ109 against *H. pylori* cultured in pH 4.5 medium was compared to bactericidal activity at pH 6.8. The plotted data are representative results from three independent experiments. The horizontal dashed line on each graph indicates the limit of detection (500 bacteria).

It is well accepted that antimicrobial activity of some drugs is affected by environmental conditions such as temperature and pH. The issue of temperature comes into play when one considers transport and storage of particular therapeutics, while pH stability is particularly important for *H. pylori* since the bacterium resides in the acidic environment of the stomach. Therefore, we tested the antimicrobial activity of SQ109 after exposure to various temperatures and acidic pH. Pre-exposure of SQ109 for 1 h to temperatures up to 95^o^C did not prevent the ability of SQ109 to kill *H. pylori*, though there was a temperature-dependent reduction in the time required to kill all of the bacterial cells (Figure 3A). SQ109 was also active at an acidic pH (pH 4.5), but again showed delayed killing kinetics as compared to activity at a more neutral pH (pH 6.8) (Figure 3B). These data suggest that although the time to kill *H. pylori* is extended by high temperatures and low pH, the drug remains bactericidal against the microbe.

### Comparison of SQ109 antibacterial activity with conventionally used anti-*H*. *pylori* antibiotics

**Figure 4:**
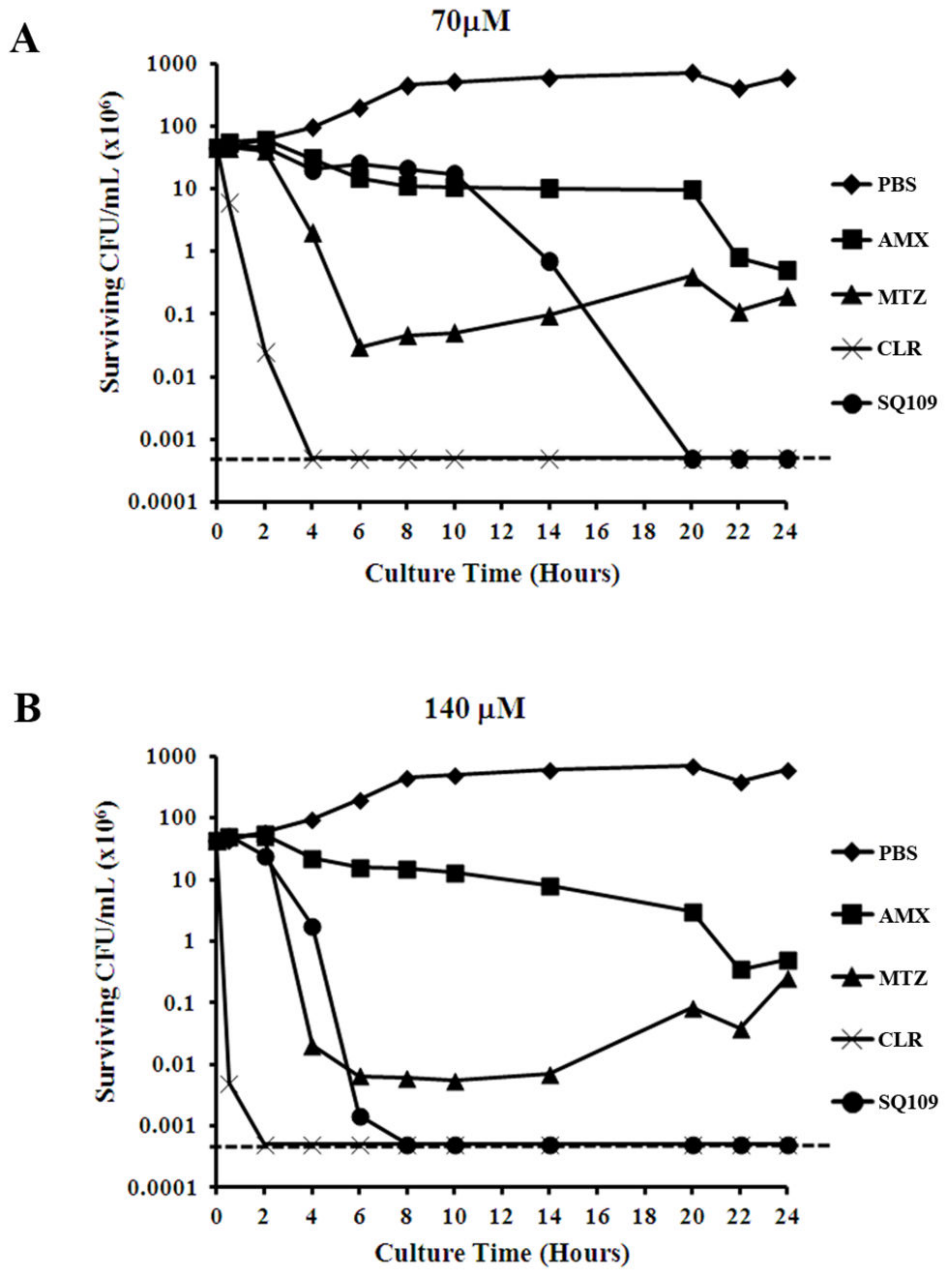
Molar-to-molar comparison of the antibacterial activity of SQ109, amoxicillin, metronidazole, and clarithromycin. Time-kill assays for two drug concentrations, 70 µM (**A**) and 140 µM (**B**), were used to compare the bactericidal activity of SQ109 to those of conventional antibiotics currently used for the treatment of *H. pylori* infection. The data are representative results from three independent experiments. The horizontal dashed line on each graph indicates the limit of detection (500 bacteria).

Any newly developed anti-*H. pylori* drug should show better or at least comparable efficacy to antibiotics currently used in treatment. We directly compared the antibacterial activity of SQ109 to equimolar concentrations of AMX, MTZ and CLR. Treatment with 70 µM of each of the drugs resulted in various rates of killing of the bacterial cells (Figure 4A). At this concentration, no viable *H. pylori* cells were recovered following 20 h of treatment with SQ109 (limit of detection = 500 bacteria). This was superior to the results obtained with AMX and MTZ, which both showed only 2-3 logs of killing in total, but inferior to CLR, which completely eliminated the bacterial culture after 4 h of treatment (Figure 4A). Increasing the concentration of the drugs to 140 µM increased the slope of the killing curve for each of the drugs, demonstrating time- and concentration-dependent effects of each drug. When the bacteria were exposed to 140 µM SQ109, no detectable CFU were observed after 8 h of exposure (Figure 4B). Once again, this antibacterial activity was superior to the results obtained with AMZ and MTZ, but the time for bacterial clearance was longer than for CLR (2 h). Taken *en masse*, these results indicate that SQ109 is superior or comparable to drugs currently used in anti-*H. pylori* therapy.

### *H. pylori* displays a low frequency of spontaneous resistance to SQ109 and further analysis suggests that surviving bacteria are largely “persisters”

Given that one of the greatest challenges posed by *H. pylori* is the development of resistance to nearly all standard drugs, we sought to examine the frequency of spontaneous resistance to SQ109. We examined this frequency for three *H. pylori* strains, G27 (most sensitive), 26695 (least sensitive), and B105 (least sensitive clinical isolate). Based on the determined MBC of 26695 (Table 1), we chose to test the frequency of resistance by plating on HBA plates containing 100 µM, 200 µM, or 400 µM SQ109. As shown in Table 3, at 100 µM SQ109, the frequency of resistance was 2.02 x 10^-11^, 2.08 x 10^-10^ and 1.9 x 10^-9^ for G27, 26695 and B105, respectively. At 200 µM SQ109, the rates were 2.13 x 10^-12^, 2.63 x 10^-11^ and < 9.6 x 10^-13^, respectively. No colonies were ever obtained on 400 µM SQ109 plates, even after testing more than 10^12^ cells.

**Table 3:** *H. pylori* frequency of resistant mutants to SQ109.

***H. pylori* Strain**	**SQ109 Plate Concentration (µM)**	**Total CFU^#^**	**Frequency of Resistance to SQ109 (Resistant CFU/Total CFU)**
G27	0	9.40E+11	ND
	100	19	2.02E-11
	200	2	2.13E-12
	400	0	< 1.06E-12
26695	0	1.94E+12	ND
	100	403	2.08E-10
	200	51	2.63E-11
	400	0	< 5.15E-13
B105*	0	1.03E+12	ND
	100	1976	1.90E-09
	200	0	< 9.63E-13
	400	0	< 9.63E-13

^#^ CFU = Colony Forming Units. ^*^ Low passage clinical isolate from a gastric ulcer patient. The data represent results from at least four independent experiments performed in triplicate plates.

The occurrence of persister cells as opposed to true resistant bacteria has been associated with enhanced antibiotic failure for many microbes. Therefore, to determine if the resistant colonies we obtained were in fact stably resistant, we utilized mutant stability tests to further analyze several of the colonies after growth in the absence of SQ109. Data for six of these colonies can be found in Figure S2. For colonies obtained from G27, which displays a MIC of 10-15 µM for SQ109, we observed a slight increase (approximately 2-fold) in the MIC for the resistant colonies. Conversely, no change in the apparent MBC was observed for these strains. Similarly, isolates obtained from B105 showed little to no difference in SQ109 MIC or MBC as compared to the parental wild-type strain. Moreover, when cultures of the resistant strains were spotted on HBA plates containing 100 µM or 200 µM SQ109, the number of colonies obtained, and thus frequencies of resistance, was identical to the wild-type strain (data not shown). These data suggest that the observed resistance was primarily transient in nature and likely originated from physiological adaptation rather than true resistance as a result of spontaneous mutation. Overall, these data demonstrate a very low frequency of resistance to SQ109 for all the strains tested, including a low-passage clinical isolate from a gastric ulcer patient.

### SQ109 bactericidal activity is not due to disruption of *H. pylori* membranes

Antimicrobial agents work through various mechanisms that may target either the bacterial cell wall and/or cytoplasmic components. Antibiotics that target bacterial membranes may directly interact with the lipid bilayer and/or structural proteins found within the membranes. Previous studies using *M. tuberculosis* demonstrated that SQ109 exerts its antibacterial activity by targeting the cell wall [32]. Thus, in order to initiate characterization of the mechanism of action of SQ109 against *H. pylori*, we analyzed the ability of the drug to permeabilize the bacterial membrane. For these studies, we utilized a neutral fluorescent dye, NPN, whose fluorescence intensity increases in hydrophobic environments such as those resulting from damaged phospholipid bilayers. NPN is known to be non-fluorescent when excluded by intact cell membranes but fluoresces in the presence of damaged membranes [38,39].

**Figure 5:**
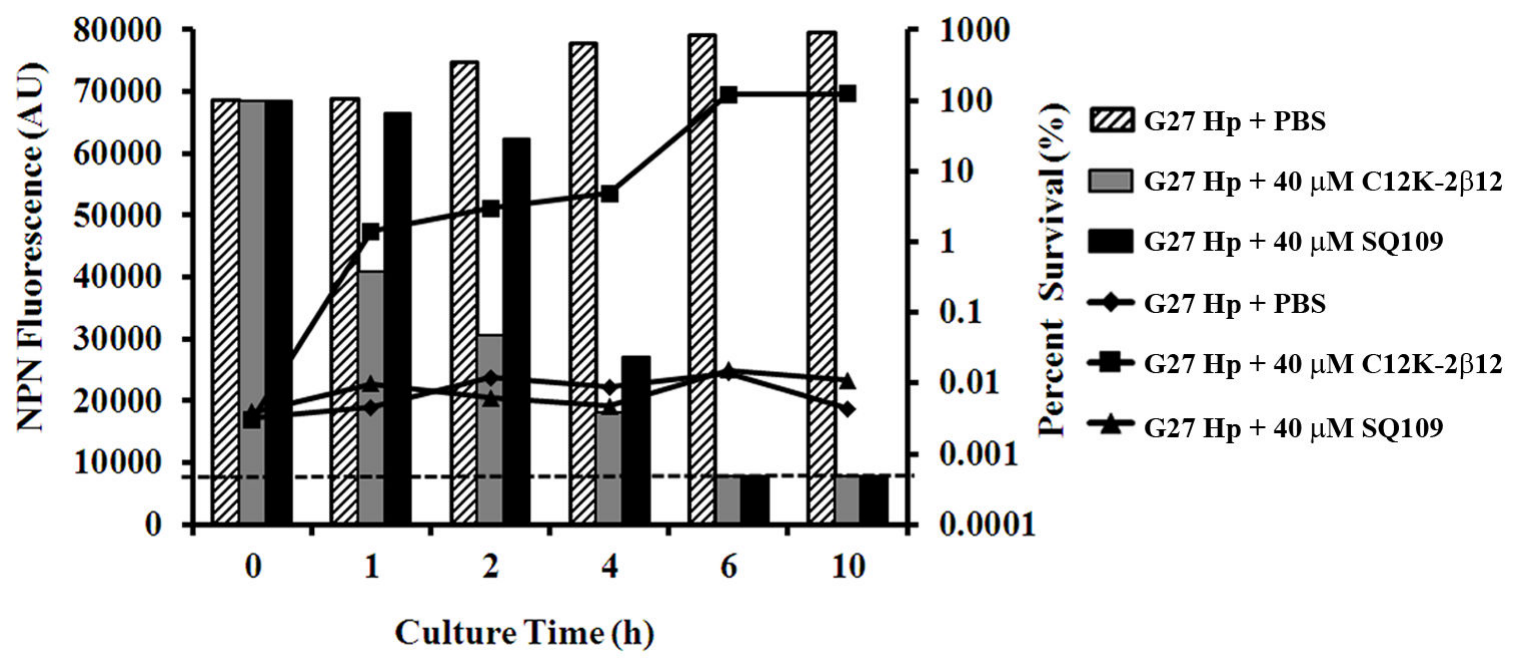
1-N-phenylnaphthylamine membrane-permeabilization assay demonstrates that SQ109 bactericidal activity is not via membrane lysis. Membrane-permeabilization and bactericidal assays were performed in parallel using *H. pylori* G27 cultured for 24 hours in the presence of 140 µM SQ109. Membrane permeation and bactericidal activity were monitored by fluorescence increase and by CFU enumeration using the micro-dilution plating method, respectively. Bacterial cells grown in the presence of 40 µM C_12_K-2β_12_ or PBS (no drugs) were used as controls. Approximately 5 x 10^6^ bacterial cells were sampled at 1 h, 2 h, 4 h, 6 h and 10 h to determine 1-N-phenylnaphthylamine (NPN) uptake and bactericidal activity. Following excitation (λ = 350 nm) and emission (λ = 420 nm), the NPN fluorescence intensity was recorded in arbitrary units (AU) as a correlate of membrane permeation. Bactericidal activity was expressed as percent survival of starting CFU. The data shown are representative results of three independent experiments. The left axis presents NPN fluorescence depicted by lines while percent survival is shown on the right axis and is indicated by bars. The horizontal dashed line indicates the limit of detection (500 bacteria).

Incubation of the G27 strain with 140 µM SQ109 resulted in no change in NPN fluorescence and showed fluorescence levels that appeared similar to PBS-treated (negative control) bacteria (Figure 5 line graphs). In contrast, samples treated with C_12_K-2β_12_ (positive control) showed an exponential increase in fluorescence; C_12_K-2β_12_ is known to cause membrane damage via the formation of pores [33]. SQ109 does not inhibit fluorescence of NPN since incubation of *H. pylori* with a combination of SQ109 and C_12_K-2β_12_ resulted in a similar increase in fluorescence intensity as that seen with C_12_K-2β_12_-treated bacteria (data not shown). Despite the lack of evidence of membrane disruption, SQ109 antibacterial activity remained potent (Figure 5 histograms). Overall, these data suggest that bactericidal activity of SQ109 may occur via interaction with a cytoplasmic target and not via disruption of the phospholipid cell membrane bilayer.

### Distinct morphological and ultra-structural modifications of SQ109-treated *H. pylori* cells as visualized with TEM

**Figure 6:**
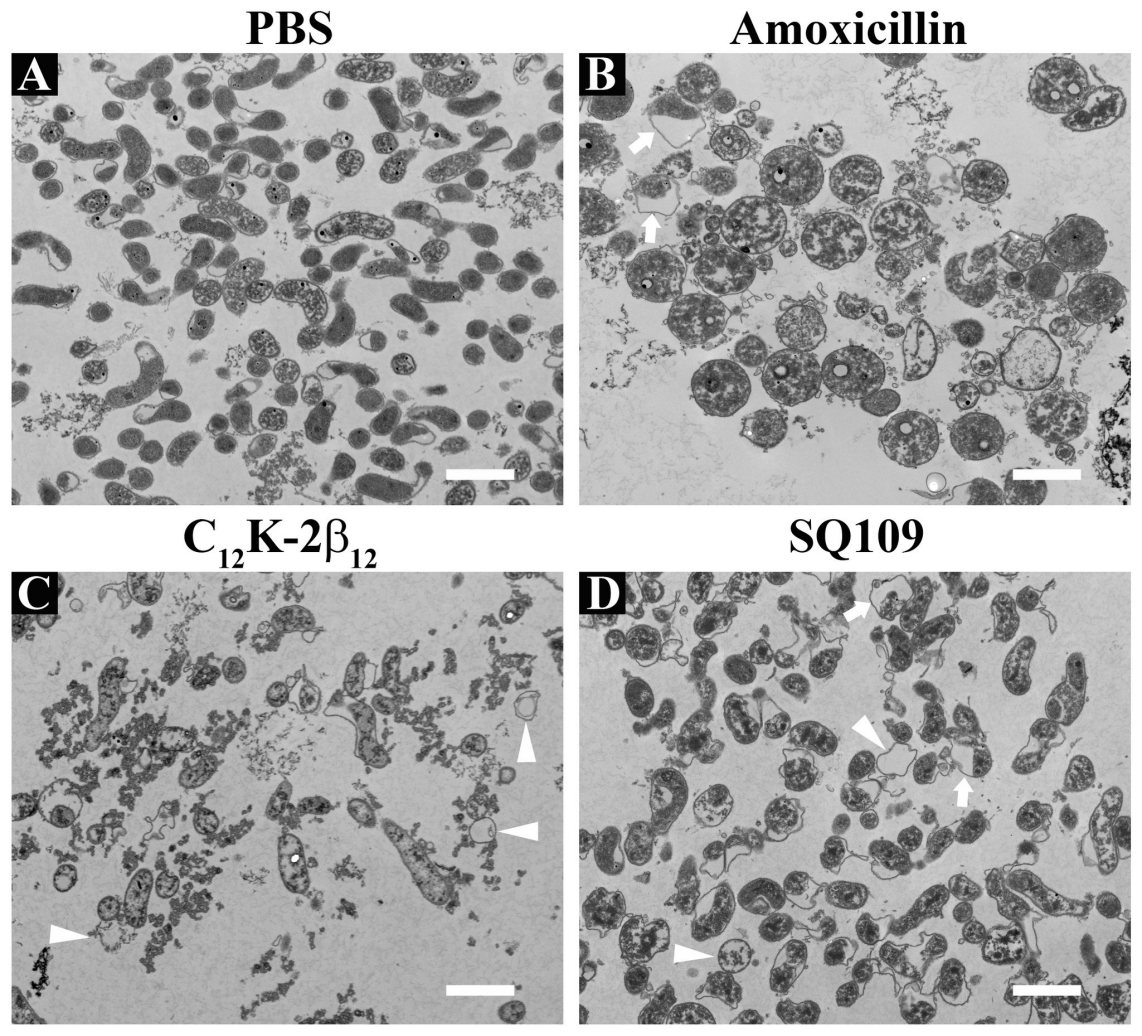
Visualization of SQ109-induced morphological changes of *H. pylori* using transmission electron microscopy (TEM). Approximately 6 x 10^7^ *H. pylori* cells were sampled following 8 h of exposure to PBS (**A**), 100 µM amoxicillin (**B**), 20 µM C _12_K-2β_12_ peptide (**C**) or 140 µM SQ109 (**D**). Cells were examined at low magnification (6,500x) on a TEM and images were acquired and processed with AMT XR60B digital camera and AMTV600 software, respectively. (**A**) demonstrates normal spiral- or comma-shaped rod morphology of *H. pylori* cells as well as intact cell membranes following treatment with PBS as a negative control drug. Amoxicillin-treated cells (**B**) show characteristically swollen cells as well as detachment of the inner membrane from the outer membrane (white arrows). C_12_K-2β_12_-treated cells (**C**) show cell lysis, numerous ghost cells (C, white arrowheads) as well as the formation of electron dense structures within the extracellular medium and inside the cells. Like AMX-treated cells, SQ109-treated cells (**D**) present with inner membrane detachment from the outer membrane (white arrows), but are not significantly enlarged. Some ghost cells are visible (white arrowheads). Scale bars = 1500 nm. The data are representative images from two independent experiments.

In *M. tuberculosis*, SQ109 inhibits cell wall biosynthesis by interfering with the assembly of mycolic acids into the cell wall core [32]. Given that the data from the NPN permeation indicated no membrane disruption of cells exposed to SQ109, we next sought to examine the effect of SQ109 on the morphology and ultra-structure of the *H. pylori* cell wall and cytoplasm using TEM. Analysis of low magnification images (6,5000x) following 8 h of exposure to SQ109 revealed that nearly all of the *H. pylori* cells (95-99%) showed a deformed coccoid morphology and contained electron-dense and distorted cytoplasmic structures (Figure 6D). In addition, a small number of apparent ghost cells (<1-2%) were visible (Figure 6D arrowheads). In contrast, the PBS-treated samples showed typical spiral-comma rod-like *H. pylori* (Figure 6A). Consistent with our previously reported data [33], positive control cells treated with AMX appeared as swollen coccoid forms or ghost cells (Figure 6B) while C_12_K-2β_12_-treated cultures showed remarkable lysis (Figure 6C).

**Figure 7:**
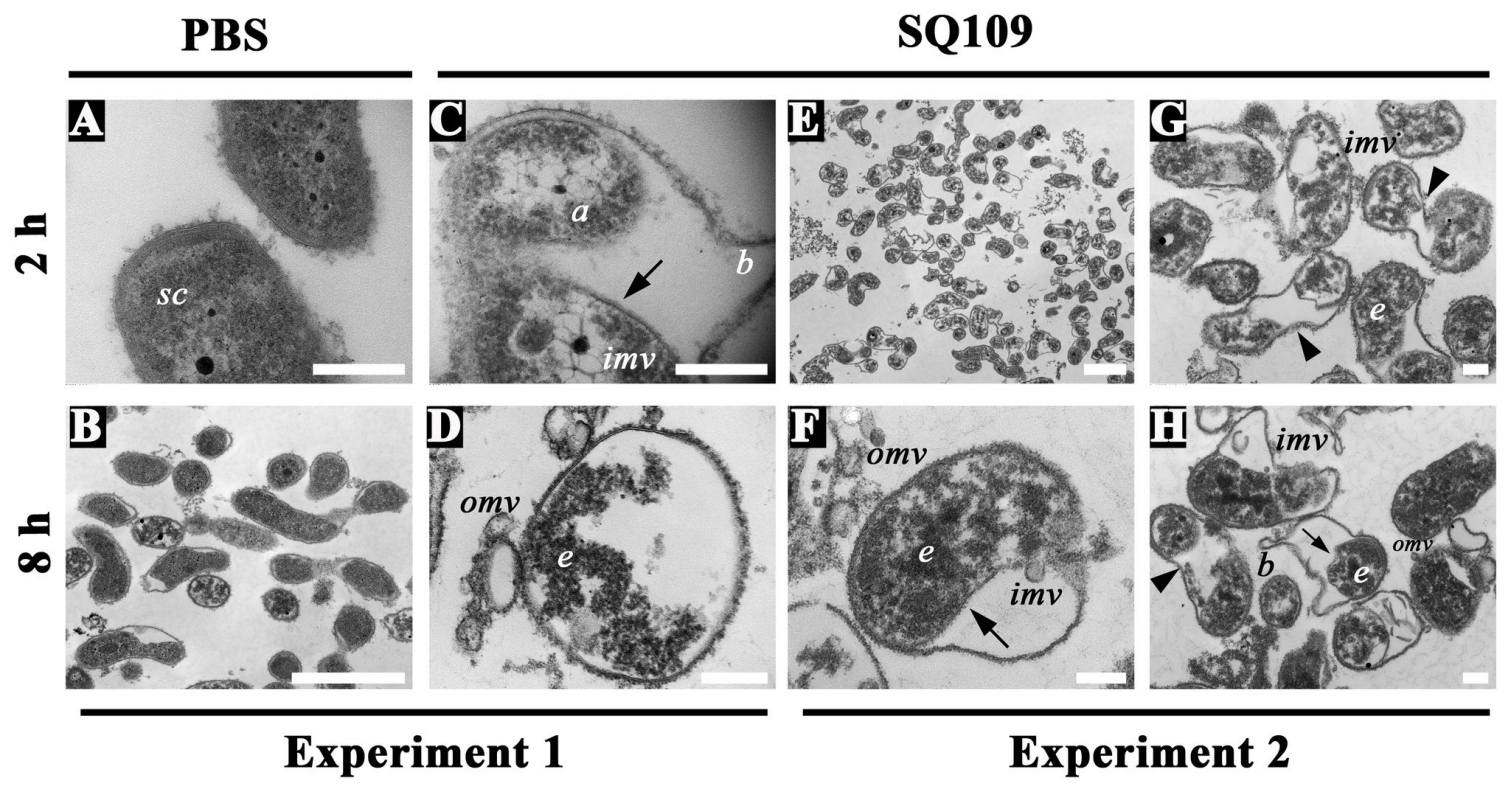
TEM analysis of SQ109-induced morphological and ultra-structural changes in *H. pylori*-cell wall, membranes, and cytoplasm. Approximately 6 x 10^7^ *H. pylori* cells were sampled following 2 h (top panels) or 8 h- (bottom panels) of culture in the presence of PBS (**A** and **B**) as a negative control or 140 µM SQ109 (C, D, **E**, **F**, **G**, and **H**). Bacterial cells cultured in the presence of 100 µM AMX or 20 µM C _12_K-2β_12_ were included as positive controls (data shown in Figure S3). PBS-treated negative control cells showed evidence of normal spiral-comma rod shaped morphology in addition to smooth homogenous cytoplasm (***sc***) and intact cell walls (**A**, **B**). In contrast, after 2 h of culture, SQ109 induced the formation of spindle actin-like cytoskeleton structures (***a***) in some cells (**C**) that appeared to condense cytoplasmic contents and lead to detachment of the IM from OM (arrows; **C**, **E**, **F**, **G**, and **H**) of nearly all cells resulting in complete disappearance of the periplasmic region; 95-99% of these cells also showed a deformed coccoid morphology (**E**, **G**, and **H**). After 2h and 8 h treatment with SQ109, the cells further showed blebs (***b***), electron-dense structures (***e***), evidence of complete loss of the IM and part of the cell wall with only the OM remaining intact (**D**), formation of two aberrant cytoplasmic bodies from a single cell (arrowheads) (**G** and **H**), and formation of outer membrane vesicles (omv) and inner membrane vesicles (imv) (**C**, **F**, **G**, and **H**). All scale bars are of 200 nm except **B** and **E** which measure 1500 nm. Representative images from two independent experiments are presented.

Little or no cell membrane disruption of *H. pylori* by SQ109 was indicated by the NPN uptake assay (Figure 5). However, morphological changes associated with SQ109 treatment (Figure 6D) contradicted the absence of membrane damage. Thus, in order to conduct a detailed analysis of the effects of SQ109 on the *H. pylori* cell wall and cytoplasmic contents, we next examined the ultra-structural features of cells treated with SQ109 for 2 h or 8 h at higher magnification (42,000x). Cells treated with PBS, AMX or C_12_K-2β_12_ peptide, were included as controls. Representative TEM results of these studies are presented in Figure 7 and Figure S3.

As shown, PBS-treated cells showed an intact cell wall with both inner (IM) and outer (OM) membranes appearing largely unperturbed. In addition, these cells tended to display a uniformly smooth bacterial cytoplasm, ***sc*** (Figure 7A and 7B, Figure S3-A, B, and C). In contrast, bacterial samples exposed to AMX showed separation of inner membranes from the outer membranes (arrows), blebs (***b***), outer membrane vesicles (omv) and ghost cells (gc) indicative of cell lysis (Figure S3-D, E and F). Like PBS-treated cells, AMX-treated bacteria typically showed uniformly smooth cytoplasm (sc). The treatment of the bacteria with C_12_K-2β_12_ revealed the formation of pores (p) and sloughing (s) in the outer membrane and electron-dense structures (e) in the cytoplasm (Figure S3-G, H and I). The results observed with AMX- or C_12_K-2β_12_-treated cells are similar to our previous data [33].

After 2 h of treatment of *H. pylori* with SQ109, there was evidence of outer membrane blebs (b) and inner membrane separation from the outer membrane to form bifocal actin-like spindle cytoskeleton structures (a) that appeared to condense the cytoplasmic contents to form inner membrane vesicles (imv) (Figure 7C, Figure S3-J). Figure 7D shows complete dissolution and separation of the inner membrane from the outer membrane with evidence of formation of outer membrane vesicles (omv) and subsequent lack of periplasm. In addition, SQ109 appeared to interact with cytoplamic target(s) as indicated by the presence of electron-dense structures (e) in the cytoplasm of nearly all the cells examined after 2 h or 8 h exposure to the peptide (Figure 7C–H, Figure S3-J–L), suggesting that SQ109 may also interact with bacterial cytoplasmic macromolecules, possibly nucleic acids and/or proteins [33]. There was also evidence of SQ109-induced disintegration of the inner membrane (Figure S3-K arrowheads) as well as an aberrant formation of two cytoplasmic bodies from a single cell (arrowheads) (Figure 7G and H, Figure S3-L) and the formation of inner membrane vesicles (imv) (Figure 7C, F, **G**, and **H**; Figure S3-J and K).

SQ109-induced changes in *H. pylori* showed both similarities and differences to AMX-or, C_12_K-2β_12_-treatment (Figure 7C–H, Figure S3-J–L). For instance, both SQ109- and AMX-treated cells showed the presence of vesicles and separation of the inner membrane from the outer membrane, but only AMX-treated cells showed a homogeneous cytoplasm. Both SQ109- and C_12_K-2β_12_-treated cells showed electron-dense cytoplasmic structures, but only C_12_K-2β_12_ induced pore formation. Overall, these results indicate differences in the mechanism of action of these drugs. Taken together, these data suggest that though the specific target is unclear, SQ109 exerts its antibacterial activity through interaction with both the inner membrane and cytoplasmic contents. In addition, the data further suggest that the mechanism of action of SQ109 is likely different from AMX and C_12_K-2β_12_.

## Discussion

The significant new finding of this study is that SQ109, an anti-tubercular drug candidate that has shown promise in the treatment of human pulmonary tuberculosis, has a strong *in vitro* efficacy against *H. pylori*. Our data show that SQ109 displays robust thermal and pH stability, induces low/no spontaneous drug resistance and shows anti- *Helicobacter* superiority over MTZ and AMX. Furthermore, the data suggest SQ109 may have a novel mode of action against *H. pylori*.

Since its discovery in the early 1980s, *H. pylori* has been causally associated with the development of a diverse number of gastric diseases [40]. Current treatment strategies require the extended use of multiple antibiotics and a PPI [41–44], which leads to contraindications in some patients. This fact, plus the cost of using multiple drugs, has resulted in patient compliance issues [45,46]. Additionally, *H. pylori* exhibits a high frequency of mutation and genetic recombination [34,35], which no doubt contributes to the rapidly emerging antibiotic resistance of *H. pylori* in many areas of the world. Moreover, while *H. pylori* is primarily considered an extracellular pathogen, a sub-population of the infecting bacterial cells can survive as intracellular bacteria [47–49]. This finding when combined with ineffectiveness of currently used antibiotics in targeting intracellular *H. pylori*, suggests that the intracellular bacterial population of cells may be partially responsible for increasing rates of treatment failure [50,51]. Thus, the combination of the existence of “intracellular persister” bacteria and the emergence and spread of antibiotic resistant strains strongly suggest that new drugs and treatment strategies are needed for *H. pylori*.

SQ109, a new anti-tubercular drug that is currently in phase 2 clinical trials for adult pulmonary TB, is safe and well-tolerated in humans, and has the capacity to achieve intracellular bactericidal concentrations that kill *M. tuberculosis* [28,29]. Moreover, the apparent resistance rate of *M. tuberculosis* against SQ109 is 2.55x10^-11^, suggesting that the occurrence of clinical resistance to this drug may not be an issue [52,53]. These exciting findings combined with the fact that SQ109 accumulates in the stomach at high concentrations following oral administration in animal models [30,54], led us to test the *in vitro* susceptibility of *H. pylori* to SQ109.

For our analysis, we utilized concentration-time-kill assays to determine the MIC, MBC and killing kinetics of SQ109 against *H. pylori*, since this methodology is superior to measuring the 24 h, 3-log decrease in CFU/ml; the latter method does not adequately predict bactericidal activity [55]. Since strain-specific susceptibility to antibiotics has previously been demonstrated [31,56,57], we included a large collection of *H. pylori* strains to mimic geographic and clinical diversity. All 26 of the *H. pylori* strains tested were susceptible to SQ109. Notably, in addition to common laboratory strains and low passage clinical isolates, our collection included a multidrug resistant clinical isolate that was unable to be cleared by successive rounds of traditional anti-*H. pylori* therapy, suggesting that SQ109 may be useful for treatment of *H. pylori* infection even if the strain in question is resistant to traditional antibiotics (Figure S1). While the relative range of susceptibility to SQ109 was relatively small, 5 of the 26 strains were slightly less susceptible to the drug. These include strains B105, B107, B289, USU102 (Table 2), and 26695 (Table 1), the last of which was also previously shown to be less susceptible to other novel classes of anti-*H. pylori* therapies that are being developed [31]. While the nature of these minor strain-specific differences in sensitivity is not clear, it is possible that they are either due to differences in the SQ109 target within each of the strains, or drug accessibility to the target.

Current work in *M. tuberculosis* indicates that SQ109 interferes with the assembly of mycolic acids into the cell wall of the bacterium [58,59]. Specifically, the drug targets MmpL3, which appears to be a transporter of the mycobacterial cell wall component trehalose monomycolate (TMM) [32]. *H. pylori* does not produce mycolic acid and does not appear to encode a close protein homolog of MmpL3; BLAST analysis of MmpL3 directly against the *H. pylori* database shows that the closest *H. pylori* homolog (E=10^-4^) is the translocase subunit SecD, which is a component of an export system responsible for translocation of some proteins across the cell membranes. Converse analysis of the *H. pylori* SecD protein directly against the *M. tuberculosis* database shows that there is a much more closely related protein (E=10^-26^) in this bacterium that is likely the functional homolog of SecD; MmpL3 is the second most closely related protein (K. Sacksteder personal communication).

The specific SQ109 target and mechanism(s) of action within *H. pylori* remains unknown. However, our data broadly suggest that SQ109 exerts its anti-bactericidal effect by a two-fold effect: first, by interacting with the bacterial cell wall to result in retraction of the inner membrane from the outer membrane, and secondly, by targeting cytoplasmic content(s) to induce formation of electron-dense structures in the cytoplasm (Figure 7F and 7G). Notably, the lack of NPN-uptake (Figure 5) suggests that the effect of SQ109 on the *H. pylori* cell wall occurs without disruption of the hydrophobic phospholipid bilayer. This phenomenon may not be unique to SQ109; similar effects in which an antibacterial agent was able to interact with bacterial membrane to gain entry into the cytoplasm without dramatic cell wall disruption have been previously reported [60]. Consistent with the lack of membrane disruption, we observed that SQ109-treated cells were not dramatically enlarged (Figures 6D and 7E); this suggests minimal or no membrane permeation induced by the drug, which is also consistent with lack of lysis. Moreover, at early time points, SQ109 induced the formation of actin-like filaments in the cytoplasm that appeared to condense the cytoplasmic contents and to cause detachment of the IM from the OM. Similar actin-like cytoskeleton structures have been reported in antibiotic-treated *Bacillus subtilis* [61], *Caulobacter crescentus* [62], and *Chlamydia trachomatis* [63]. Indeed, in studies in which antibiotics targeted MreB, which is a rod shape-determining protein linked to penicillin-binding protein-2 in bacilli that is responsible for bacterial actin-like cytoskeleton structures, the cells changed shape [63]. This finding is consistent with our observation that SQ109 induced coccoid morphology and detachment of the IM from the OM; these changes were similarly evident in AMX-treated cells. AMX exerts its effect by inhibition of assembly of cell wall peptidoglycan through the binding of PBP. Therefore, SQ109-induced detachment of IM from OM may be due to inhibition of cell wall biosynthesis, resulting in a lack of peptidoglycan, the matrix that cross-links the two membranes and keeps them together [64,65]. The differences in SQ109-induced changes as compared to those of AMX (such as the presence of electron-dense structures) and those of C_12_K-2β_12_ such as the lack of pore formation, likely relate to differences in mechanism of action. Thus, further studies are required to identify the specific target(s) of SQ109 in *H. pylori*.

Of note, many antibiotics fail in clinical trials due to an inability to reach sufficient concentrations in infected tissue. This phenomenon, which is also referred to as “first-pass effect”, has been attributed to metabolism of orally administered drugs by gastrointestinal and hepatic enzymes; this results in a significant reduction of the amount of un-metabolized drug reaching the systemic circulation [66]. However, in *H. pylori* infections, the bacterium colonizes the stomach, thus, the “first-pass effect” can be bypassed, and increased bioavailability of a drug can be achieved by oral administration of many drugs. Moreover, SQ109 has the ability to translocate into the host cell and kill intracellular *M. tuberculosis*, whereas conventional antibiotics used to treat *H. pylori* infection likely do not reach suitable concentrations inside the host cell to sterilize the environment [67,68]. Thus, SQ109 may also be effective in killing intracellular *H. pylori* [47–49], which are believed to be partially responsible for treatment failures through reseeding the infection to the gastric mucosa [47,67,68].

Prospectively, assuming a mouse stomach mass of 200 mg and the previously reported stomach tissue analysis of SQ109 concentration at 1 h and 4 h post-oral administration [30], our results indicate that the SQ109 concentration in the stomach would be approximately two- to five-fold higher than the determined drug MBC (50-100 µM; Tables 1 and 2). It is envisaged that the high concentration of SQ109 maintained for 1-4 h would give the drug time to diffuse into the mucus and/or be absorbed systemically to reach the bacterium via the basolateral surface. Thus, given the observation that the concentration of SQ109 that accumulates in the stomach of animals orally dosed with the drug is well above the MBC for *H. pylori*, it may be possible to use SQ109 as an effective monotherapy for *H. pylori* infection. This notion is further supported by our findings that the frequency of resistance is very low (Table 3) and that the antimicrobial activity of SQ109 was superior to the activity of MTZ and AMX on a molar-to-molar basis (Figure 4). Importantly, even if the drug is ineffective as a monotherapy, our finding that a normally bacteriostatic concentration of SQ109 shows synergistic bactericidal activity when administered with Cm suggests that the drug may be effective when co-administered with Cm or other clinically relevant bacteriostatic agents. Our data further describe a preliminary mechanism of action and suggest that the SQ109 acts by interaction with both cell wall and cytoplasmic contents. Thus, our data suggest that SQ109 may represent a powerful new class of drugs against *H. pylori* that may be used to significantly improve treatment success and eradication of this pathogen.

## Supporting Information

Figure S1 Determination of antibiotic resistance of *H. pylori* clinical isolate, USU103 to selected conventional antibiotics. Bactericidal assay of selected antibiotics, AMX, MTZ, CLR, and TET to the clinical isolate (USU103) was performed to analyze the resistance levels of the bacteria. The percent survival (vertical axis) of *H. pylori* clinical isolate, USU103, is shown at the indicated antibiotic concentration (horizontal axis). The bacterial CFU/ml was determined by plating following culture for 24 h in the presence of a range of concentrations of AMX (0.5-1000 µg/ml), MTZ (0.5-1000 µg/ml), CLR (0.5-100 µg/ml), and TET (0.5-100 µg/ml). Percent survival was calculated using the formula: % survival = CFUt_24_/CFUt_0_ × 100, where CFUt_0_ represents CFU at the beginning of the experiment and CFUt_24_ represents CFU at 24 h of exposure to the antibiotic. Resistance was defined as follows: for AMX (< 1 µg/ml), MTZ (< 8 µg/ml), CLR (< 1 µg/ml), and TET (< 4 µg/ml). The dotted horizontal line represents the MIC. Data are representative of three independent experiments.
(TIF)

Figure S2 Determination of mutant stability to insure absence of persisters. A total of six mutants, three from G27 (R1S1, R1S2, and R1S3) and another three from B105 (R870S1, R870S2, and R870S3) were cultured from stocks in a total of five rounds of 24 h-passages by inoculation onto SQ109-free plates. A new bactericidal assay expressed as percent survival was performed to determine MIC and MBC for SQ109 as described and defined in Materials and Methods. The mutant and parental (wild-type) MICs and MBCs were compared to evaluate the presence or absence of persisters. The data are representative of two experiments.
(TIF)

Figure S3 TEM analysis of SQ109-induced morphological and ultra-structural changes in *H. pylori*-cell wall, membranes and cytoplasm. Approximately 6 x 10^7^ *H. pylori* G27 strain cells were sampled following 2 h (upper panels) or 8 h- (middle and bottom panels) culture in the presence of PBS, no antibiotics (**A**, **B**, and **C**) as negative control, 100 µM AMX (**D**, **E**, and **F**) as positive control, 20 µM C_12_K-2β_12_ (**G**, **H**, and **I**) as positive control, or 140 µM SQ109 (**J**, **K**, and **L**). AMX-treated cells (**A**, **B**) showed detachment of inner membrane from outer membrane (double arrows) in addition to formation of vesicles (***v***), outer membrane vesicles (omv), (**E**), and ghost cells (gc), (**F**). Like PBS-treated control cells, AMX-treated cells also had smooth homogeneous cytoplasm (***sc***), (**D**, **E**, **F**). C_12_K-2β_12_ induced the formation of pores (***p***), membrane sloughing (***s***), and formation of electron-dense structures (***e***) in the cytoplasm (**G**, **H**, **I**). In contrast, at 2 h of culture SQ109 induced the formation of spindle actin-like cytoskeleton structures (***a***) that appear to condense cytoplasmic contents leading to detachment of IM from OM (double arrows) similar to AMX-treated cells. At 8 h of exposure to SQ109, cells showed blebs (***b***) that appeared to pinch off to form outer membrane vesicles (omv) (**L**) in addition to evidence of formation of inner membrane vesicles (imv) (**K**), disintegration of IM (arrowheads) and aberrant constriction of cell wall to form two cells (**L**). Scale bars (white) = 200 nm. The data are representative images from two independent experiments.
(TIF)
