# Supplementary figures and images for: In Vitro Characterization of the Anti-Bacterial Activity of SQ109 against Helicobacter pylori
Source: PLoS One. 2013 Jul 25;8(7):e68917. doi: 10.1371/journal.pone.0068917 (PMC3723868; doi:10.1371/journal.pone.0068917)

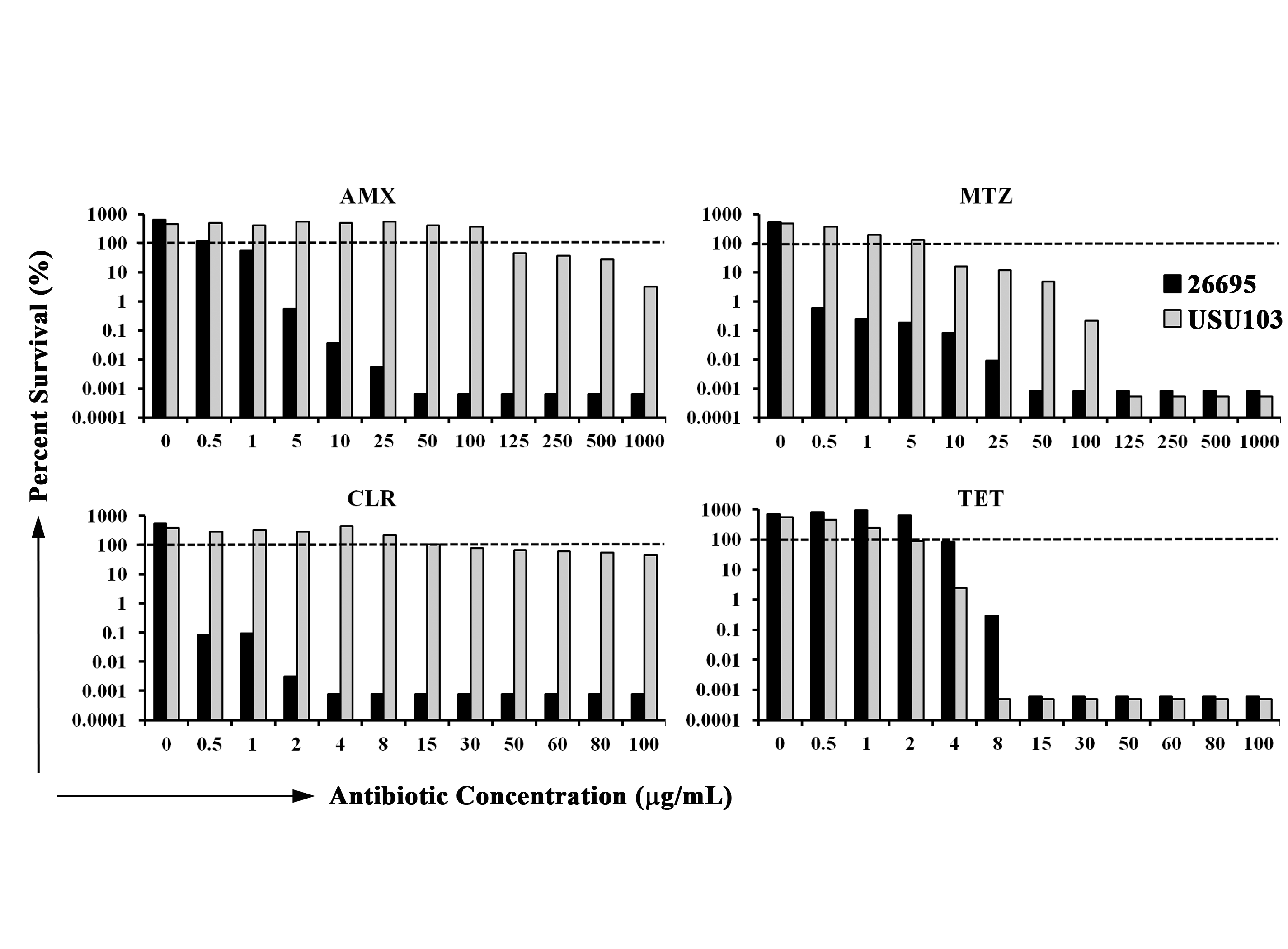

Supplement: Figure S1 — Bactericidal assay of selected antibiotics, AMX, MTZ, CLR, and TET to the clinical isolate (USU103) was performed to analyze the resistance levels of the bacteria. The percent survival (vertical axis) of H. pylori clinical isolate, USU103, is shown at the indicated antibiotic concentration (horizontal axis). The bacterial CFU/ml was determined by plating following culture for 24 h in the presence of a range of concentrations of AMX (0.5-1000 µg/ml), MTZ (0.5-1000 µg/ml), CLR (0.5-100 µg/ml), and TET (0.5-100 µg/ml). Percent survival was calculated using the formula: % survival = CFUt24/CFUt0 × 100, where CFUt0 represents CFU at the beginning of the experiment and CFUt24 represents CFU at 24 h of exposure to the antibiotic. Resistance was defined as follows: for AMX (< 1 µg/ml), MTZ (< 8 µg/ml), CLR (< 1 µg/ml), and TET (< 4 µg/ml). The dotted horizontal line represents the MIC. Data are representative of three independent experiments. (TIF) [file pone.0068917.s001.tif]

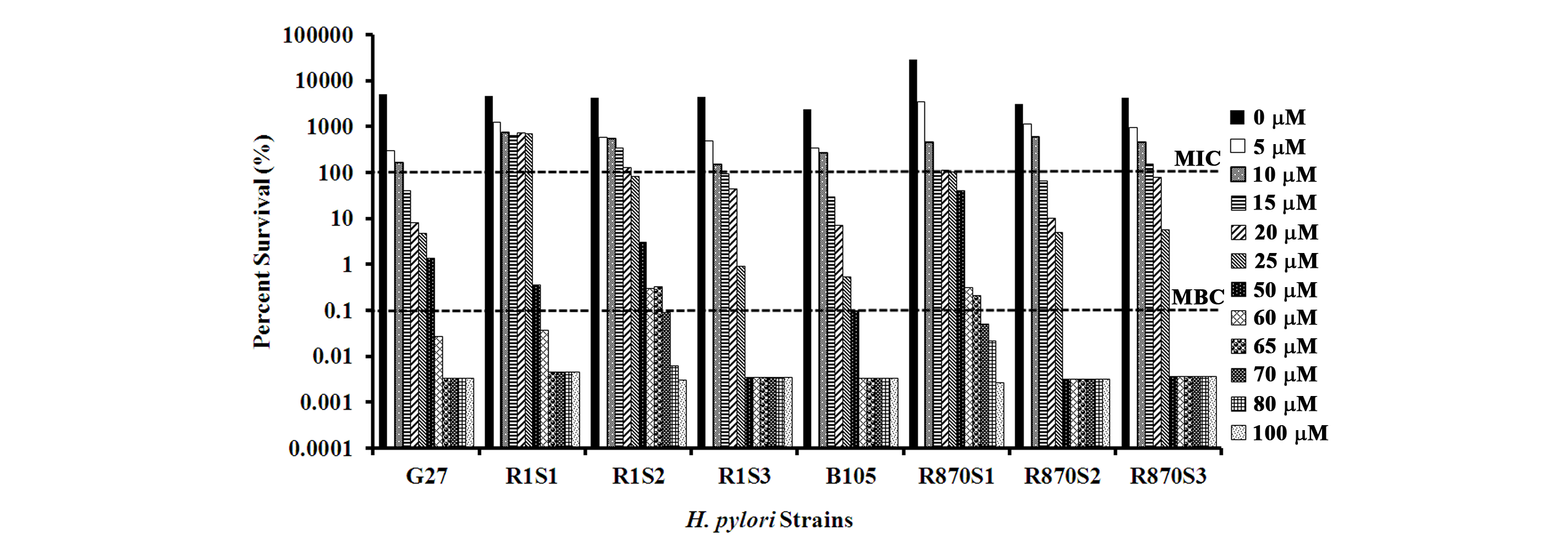

Supplement: Figure S2 — A total of six mutants, three from G27 (R1S1, R1S2, and R1S3) and another three from B105 (R870S1, R870S2, and R870S3) were cultured from stocks in a total of five rounds of 24 h-passages by inoculation onto SQ109-free plates. A new bactericidal assay expressed as percent survival was performed to determine MIC and MBC for SQ109 as described and defined in Materials and Methods. The mutant and parental (wild-type) MICs and MBCs were compared to evaluate the presence or absence of persisters. The data are representative of two experiments. (TIF) [file pone.0068917.s002.tif]

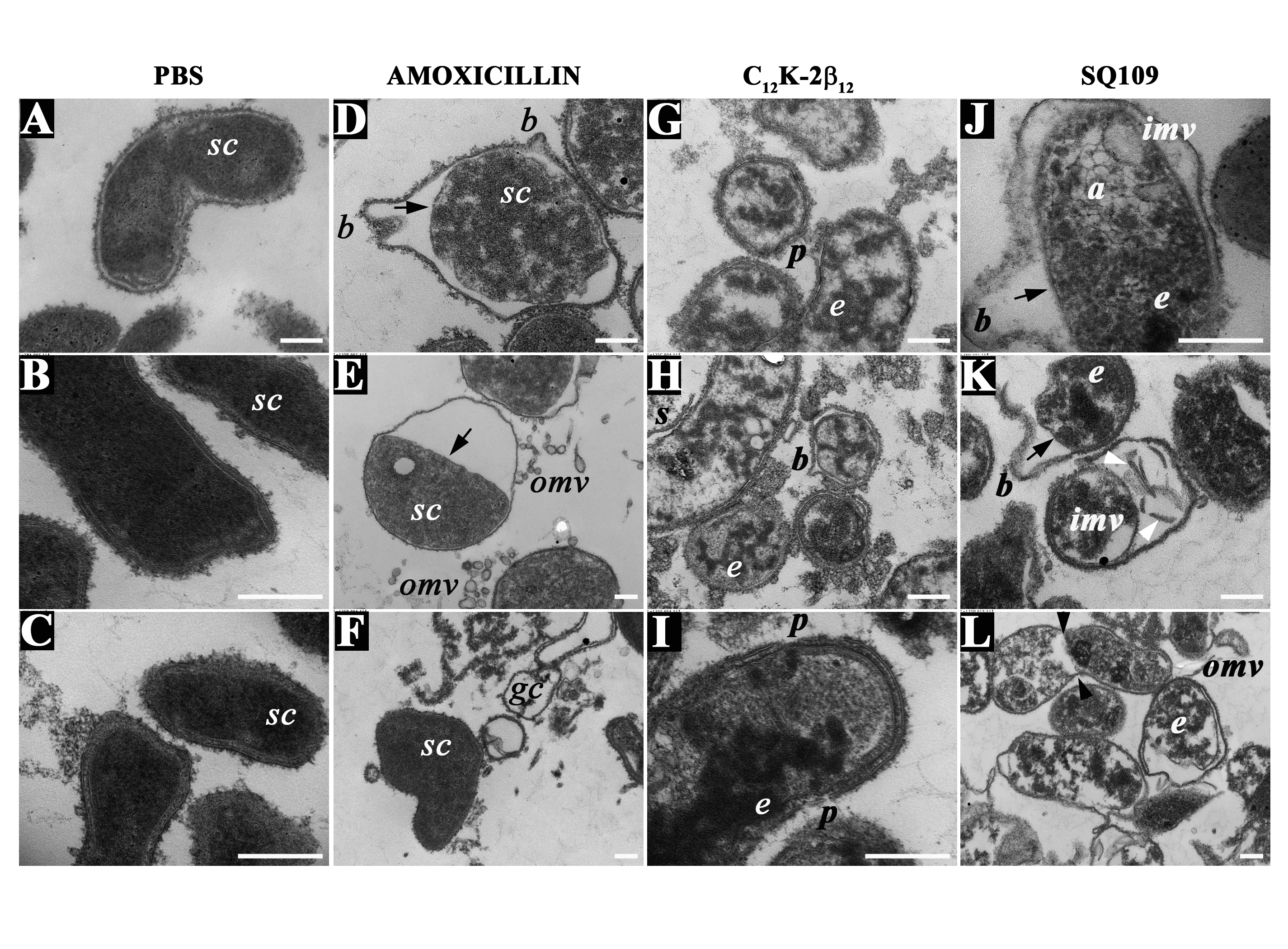

Supplement: Figure S3 — Approximately 6 x 10^7^ H. pylori G27 strain cells were sampled following 2 h (upper panels) or 8 h- (middle and bottom panels) culture in the presence of PBS, no antibiotics (A, B, and C) as negative control, 100 µM AMX (D, E, and F) as positive control, 20 µM C12K-2β12 (G, H, and I) as positive control, or 140 µM SQ109 (J, K, and L). AMX-treated cells (A, B) showed detachment of inner membrane from outer membrane (double arrows) in addition to formation of vesicles (v), outer membrane vesicles (omv), (E), and ghost cells (gc), (F). Like PBS-treated control cells, AMX-treated cells also had smooth homogeneous cytoplasm (sc), (D, E, F). C12K-2β12 induced the formation of pores (p), membrane sloughing (s), and formation of electron-dense structures (e) in the cytoplasm (G, H, I). In contrast, at 2 h of culture SQ109 induced the formation of spindle actin-like cytoskeleton structures (a) that appear to condense cytoplasmic contents leading to detachment of IM from OM (double arrows) similar to AMX-treated cells. At 8 h of exposure to SQ109, cells showed blebs (b) that appeared to pinch off to form outer membrane vesicles (omv) (L) in addition to evidence of formation of inner membrane vesicles (imv) (K), disintegration of IM (arrowheads) and aberrant constriction of cell wall to form two cells (L). Scale bars (white) = 200 nm. The data are representative images from two independent experiments. (TIF) [file pone.0068917.s003.tif]
